# *Helicobacter pylori* eradication rate of standard triple therapy and factors affecting eradication rate at Bahir Dar city administration, Northwest Ethiopia: A prospective follow up study

**DOI:** 10.1371/journal.pone.0217645

**Published:** 2019-06-04

**Authors:** Endalew Gebeyehu, Desalegn Nigatu, Ephrem Engidawork

**Affiliations:** 1 Department of Pharmacology, College of Medicine and Health Sciences, Bahir Dar University, Bahir Dar, Ethiopia; 2 Department of Pharmacology and Clinical Pharmacy, School of Pharmacy, College of Health Sciences, Addis Ababa University, Addis Ababa, Ethiopia; 3 Department of Internal Medicine, College of Medicine and Health Sciences, Bahir Dar University, Bahir Dar, Ethiopia; Cleveland Clinic, UNITED STATES

## Abstract

**Background:**

Eradication of *Helicobacter pylori* infection with standard triple therapy has been accepted to curb associated risks of chronic gastritis andpeptic ulcer disease.

**Objective:**

To assess *H*. *pylori* eradication rate of standard triple therapy and patient related factors affecting eradication rate.

**Methods:**

A facility based prospective follow up study was conducted in Bahir Dar City Administration, Ethiopia, on consented outpatients presented with gastritis and peptic ulcer disease and positive for *H*. *pylori* stool antigen test from May 2016 to April 2018. Eradication was confirmed with stool antigen test made after 4–6 weeks of standard triple therapy, comprising of proton pump inhibitor, clarithromycin and amoxicillin. Pre-developed questionnaire and data collection formats were used to collect variables before and after therapy. Bivariate and backward stepwise multivariate logistic regression was used to analyze data. P-value < 0.05 at 95%CI was considered as significant.

**Results:**

The overall *H*. *pylori* eradication rate was 90.3% (379/421). Almost 80% of the patients were urban residents. Mean (±SD) age and body weight of patients were 30.63 (± 10.74) years and 56.79 (± 10.17) kg, respectively. Self-reported adverse drug effects and area of residence of patients were factors affecting eradication rate significantly. Patients with no self-reported adverse drug effect were 3.85 (AOR: 3.85; 95%CI (1.41–5.26)) times more likely to eradicate *H*. *pylori* infection compared to those reported adverse effects. Patients living in rural area were 2.7 (AOR: 2.7; 95%CI (1.19–20.0)) times more likely to achieve eradication compared to urban residents.

**Conclusion:**

*H*. *pylori* eradication rate is within the recommended level for clinical practice, indicating that modifications of the standard triple therapy observed in the different healthcare institutions are not evidence-based. Emphasis should be given to adverse drug effects of medications and tailored counseling based on area of residence could have a contribution in improving eradication rate.

## Introduction

*Helicobacter pylori* infection remains one of the most common chronic bacterial infections affecting humans with prevalence rates varying widely among different geographical regions and ethnic groups [1, 2]. *H*. *pylori* infection causes chronic gastritis and is associated with an increased risk of upper gastrointestinal diseases, such as peptic ulcer disease, gastric cancer, and mucosa-associated lymphoid tissue lymphoma [3,4]. Eradication of *H*. *pylori* thus decreases the risk for gastrointestinal disease and is important to promote public health, especially in areas with high *H*. *pylori* prevalence.

Proton pump inhibitor (PPI)-based triple therapy is the mainstay therapy for eradication of *H*. *pylori* infection, although the regimens differ in combination of the antimicrobials used and duration of therapy. Specifically, PPI-based triple therapy, usually consisting of a PPI, amoxicillin, and clarithromycin, is a widely recommended regimen for *H*. *pylori* treatment in areas where clarithromycin resistance is low [5–8]. Accordingly, this regimen continues to be the recommended first-line treatment for *H*. *pylori* in Ethiopia. Its eradication rate has, however, not been studied. Moreover, the practice of sequential therapy is not uncommon in some healthcare institutions within the region, without supporting evidence.

An accumulating body of evidence also indicates that patient related factors, including poor compliance and antibiotic resistance are associated with eradication failure [9–11]. However, the relationship between eradication failure and other factors related to socio-demographic and clinical characteristics of patients are still a subject of controversy [12–14] and need further investigation. In addition, it is not uncommon to use some food items either alone or in combination with the triple therapy, usually in people with complaint of acid pepsin disorder. The common food items used in the study area are mucilage of Flaxseed or Linseed (*Linum Usitatissimmum*) and Fenugreek (*Trigonella foenum*-*graecum*), which are known as Telba and Abish, respectively, in the local Amharic language. Although there are several nutritional and animal studies supporting the protective or healing effect of these plants in gastritis and peptic ulcer [15–19], there are no studies done on the effect of these food items on eradication rate of *H*. *pylori*. Thus, the present study sought to address such issues that appropriate measures could be taken in the fight against *H*. *pylori* infection.

## Methods

### Ethics statement

The study was approved by the Institutional Review Board of College of Medicine and Health Sciences, Bahir Dar University (Reference No: BCS/171/08). Permission was sought from the health institutions after presentation of the ethical approval. All the drugs used in eradication therapy were approved by Food, Medicine, Healthcare Administration and Control Authority (FMHACA) of Ethiopia and the treatment protocol is as per the national General Hospital Guideline. Patients were informed about the benefits and risks of the study as well as their full right to withdraw from the study at any time in point without jeopardizing the care they receive from the health facility. Moreover, privacy and confidentiality were maintained through anonymity and restricting data access S1 File.

### Study design and setting

A facility based prospective follow up study was conducted from May 2016 to April 2018 in Bahir Dar, the capital city of Amhara Regional State, located 565 kilometers Northwest of Addis Ababa, the capital of Ethiopia. The study was conducted at two healthcare institutions, Adinas General Hospital and Kidanemihret Higher Clinic, which were selected based on the practice of using stool antigen test for diagnosis of *H*. *pylori*.

### Patients and eradication therapy

The study was conducted on 421 *H*. *pylori* positive patients living in rural and urban settings. Volunteer adult (age ≥18 years) outpatients, who had agreed to give written consent and willing to come back following completion of triple therapy (4–6 weeks) for checking eradication were included in the study. Those who were seriously sick or referred from other facilities as well as those who do not speak the local language (Amharic) were excluded from the study. There were two encounters with the patients: the first was at the time of diagnosis, a time when they were recruited, and the second was at the completion of eradication therapy.

Both primary diagnosis as well as eradication of *H*. *pylori* after 4–6 weeks therapy was confirmed by a stool antigen test, which is recommended by both European and Japanese guidelines [20]. Stool antigen test has the advantage of being a direct and noninvasive test because it detects either the bacteria or part of it (DNA, antigen) in an easily obtained specimen. Review studies reported sensitivity and specificity of the test to range from 88.8%-91.8% and 94.1%-94.5%, respectively [21,22]. Collection of stool sample was made with stool cup. The collected sample was immediately delivered to the respective clinical laboratory of the healthcare institutions and laboratory testing was conducted according to the Manufacturer’s recommendation (SD BIOLINE *H*. *pylori* Ag, Standard Diagnostics, Inc. Korea). *H*. *pylori* positive adult outpatients were treated by internists with a PPI-based triple therapy: omeprazole 40 mg or pantoprazole 40 mg, twice/day for 15 to 30 days); clarithromycin (500 mg), and amoxicillin (1000 mg), each twice/day for 10 or 14 days.

### Data collection and management

Structured questionnaire developed from the literature was used to collect data in both the recruitment and follow up phases S1–S4 Tables. Patients’ sociodemographic and medical information was collected during the first encounter and data on drug utilization, adverse drug effects, and added-on therapy (homeopathic medicines) was collected during the follow up period. Phone calls were made to most patients to remind their appointment period.

Pre-test of the questionnaire was done on 5% of the sample size in another healthcare institution in the study area to ensure whether the questionnaire was able to capture the required information and modifications were made accordingly. Data was collected by trained clinical pharmacists and nurses. Data accuracy and consistency was assured by the study team on daily basis through direct supervision of data collectors and inspection of collected data.

On completion of therapy, stool sample was collected to determine the success or failure of *H*. *pylori* eradication therapy. Therapy was considered as successful on having negative stool antigen test following 4 to 6 weeks of eradication therapy, whereas positive result was taken as eradication failure. The latter patients were made to consult their physician for further evaluation and treatment.

### Data analysis

Data were entered and analyzed using SPSS statistical package version 21.0. Descriptive statistics such as percentages, means and standard deviations were used to describe data. Chi-square test was used to assess failure-success differences in *H*. *pylori* eradication. Bivariate and multivariable logistic regressions were used to identify predictors of failure of eradication using triple-therapy. The Hosmer-lemeshow test was checked to assess the model fitness to conduct logistic regression. Each variable which fulfilled the Hosmer-lemeshow test on binary logistic regression (variable with no significant difference between observed and expected values) was retained for multivariable logistic regression. Backward stepwise logistic regression model was used during multivariable logistic regression to control confounding effect. Odds ratio with 95% confidence intervals was calculated for each of the independent variables using P-value < 0.05 as the level of significance.

## Results

### Sociodemographic characteristics

From a total of 526 consented patients, 421 were able to come back to the healthcare institution for stool antigen test after eradication therapy. As shown in Table 1, the mean age (SD) of patients was 30.63 (± 10.74) years, which ranged from 18–86. More than 70% of patients were under 35 years old. The mean weight of patients was 56.71 (±10.19) kg and the mean body mass index was 21.09 (±4.16).

**Table 1:** Physical characteristics of *Helicobacter pylori* infected patients (n = 421).

Variable	Minimum	Maximum	Mean	Std. deviation
Age	18	86	30.63	10.14
Height	1.45	1.90	1.64	0.07
Weight (kg) before therapy	37	93	56.72	10.19
Weight (kg) after therapy	37	90	56.79	10.18
BMI before therapy	13.84	31.63	20.92	3.26
BMI after therapy	13.75	32.05	20.96	3.31

As shown in Table 2, two-third of the patients were females and majority (80%) of them were urban dwellers. Close to two-third (63.4%) of them were married and a sizable proportion (42%) of them attended college education or above. Around 38% of the patients were employees of government and private sectors with monthly paid salary and the rest were engaged in own income generating activities. Majority (86%) of the patients were followers of Ethiopian Orthodox Church.

**Table 2:** Socio-demographic characteristics *Helicobacter pylori* infected patients attending in selected healthcare institutions at Bahir Dar City Administration, May 2016 to April 2018 (n = 421).

Variables and Categories		Frequency and percentage	Eradication
Variable	Variable categories		Rate in %
Sex	Male	145(34.4)	89.7
	Female	276(65.6)	90.2
Age	18–24	125(29.7)	88.8
	25–34	172(40.9)	90.7
	35–44	75(17.8)	90.7
	≥45	49(11.6)	89.9
Residence	Urban	336(79.8)	88.1
	Rural	85(20.2)	96.5
Religion	Orthodox	362(86.0)	90.1
	Muslim	53(12.6)	88.7
	Protestant	6(1.4)	100
Zonal address	BD city admonition	169(40.1)	90.5
	West Gojjam	96(22.8)	94.8
	South Gondar	62(14.7)	87.1
	Awi zone	52(12.4)	84.6
	Others*	42(10.0)	88.1
Marital status	Single	145(34.5)	91.7
	Married	267(63.4)	89.5
	Divorced/Widowed	9(2.1)	77.8
Occupation	Employee	159 (37.8)	90.6
	Non-employee	262(62.2)	89.7
Educational status	Primary education (1–8) and below	141(33.5)	93.6
	Secondary education (9-12^th^grade)	104(24.7)	84.6
	Attended college and above	176(41.8)	90.3

*Others include: East Gojjam, North Gondar, and Metekel

### Medical information and *H*elicobacter *pylori* eradication rate of patients

Medical information and *H*. *pylori* eradication rate of standard triple therapy of the study participants is summarized in Table 3. More than half (56%) of the patients confirmed that they had visited other healthcare institutions for seeking medical care before current eradication therapy, but they did not know the type of therapy they had taken. Almost 85% of them said that they had been living with their gastrointestinal complaint for more than a month. Almost half (50.5%) of the patients responded that they had felt pain after meal, while 29% reported that the pain feeling persisted throughout the day. About a fourth (25.6%) of them were living with chronic diseases and 56.3% were taking alcohol before initiation of the triple therapy.

**Table 3:** Medical information and *Helicobacter pylori* eradication rate of standard triple therapy among patients in selected healthcare institutions at Bahir Dar City Administration, May 2016 to April 2018 (n = 421).

Variables and Categories		Frequency and percentage	Eradication Rate in %
Variable	Variable categories		
Outcome of triple therapy	Eradication succeeded	379 (90.02)	90.02
	Eradication failed	42(9.98)	9.98
Time elapsed since gastritis started	< 1 month	67(15.9)	92.5
	>1 to 3 months	116(27.6)	91.4
	> 3 months	238(56.5)	88.7
Previous history of gastritis therapy	Yes	235(55.8)	88.9
	No	186(44.2)	91.4
Presence of other chronic disease(s)	Yes	108(25.6)	89.7
	No	313(74.3)	90.7
Self-reported alcohol intake	Yes	237(56.3)	90.3
	No	184(43.7)	89.7
Pain feeling period in the day	After meal	217(51.5)	90.3
	Persistent in the day	122(29.0)	88.5
	Long interval between meals	82(19.5)	91.5
Use of Flaxseed or Fenugreek	Yes	135(32.1)	86.7
	No	286(67.9)	91.5
Triple therapy regimen durations	10 day Amoxa + Clari + PPI	279(66.3)	89.8
	14 day Amoxa + Clari + PPI	142(33.7)	91.5
Self-reported adverse drug effects	Yes	110(26.1)	81.8
	No	311(73.9)	92.9

Almost a third (32.1%) of the patients reported that they had taken diets traditionally believed to have healing effect on gastritis and peptic ulcer disease like Fenugreek and Flaxseed together with the triple therapy. These diets appeared to be commonly used by urban (34.2%) than rural (23.5%) patients. Almost a fourth (26.1%) of the patients responded that they had experienced adverse drug effects while taking medications and were unable to take their medication properly. Commonly reported adverse effects included, among others, gastrointestinal upset (16.4%) (nausea and vomiting, diarrhea, indigestion, and change in bowel habit) and headache (3.5%). Self-reported adverse drug effects were more in urban patients (28.3%) than rural ones (17.5%). Self-reported adverse drug effects among patients who used either Fenugreek or Flaxseed was comparable with patients that had not used these diets (25.9% vs. 26.2%).

The overall *H*. *pylori* eradication rate (success of therapy) was 90.02%. Pantoprazole (73.2%) was the most commonly used PPI over omeprazole (26.6%). Nearly two-third (66.3%) of the patients received PPI-based triple therapy for a duration of 10 days, whereas the remaining took for 14 days. No significant difference was noted in both *H*. *pylori* eradication rate and development of adverse drug effects between patients receiving therapy for 10 and 14 days.

The mean age of patients on whom *H*. *pylori* eradication was successful and unsuccessful was 30.64 (±10.72) and 30.63 (±10.87), respectively. The mean weight of patients before and after eradication therapy was 56.71 (±10.19) and 56.79 (+10.17) kg, respectively.

### Factors associated with *H*. *pylori* eradication rate

Bivariate and multiple logistic regression analysis is depicted in Table 4. On bivariate logistic regression analysis, three variables: rural residence (COR: 5.61, 95%CI (1.33–23.69), p = 0.019); secondary educational status (COR: 0.38, 95CI (0.16–0.89), p = 0.025); and self-reported adverse drug effects (COR: 2.92 95%CI (1.52–5.59); p = 0.001) were significantly associated with failure of *H*. *pylori* eradication therapy. On multivariable logistic regression model analysis, rural residence and self-reported adverse drug effects were factors affecting eradication therapy. Those patients living in rural area were 2.7 (AOR: 2.7; 95% CI (1.19–20.0) (p = 0.032)) times more likely to eradicate *H*. *pylori* infection with triple therapy compared to those living in urban area. Patients without adverse drug effect were 3.85 (AOR: 3.85; 95% CI (1.41–5.26) (p = 0.002)) times more likely to eradicate *H*. *pylori* infection with triple therapy compared to those patients with adverse effects.

**Table 4:** Logistic regression analysis of factors associated with *H*. *pylori* eradication triple therapy in selected healthcare institutions at Bahir Dar City Administration, May 2016 to April 2018. (n = 421).

Variables and Categories		SAT after HPET *		Crude odds ratio	Adjusted odds ratio (CI = 95%)
Variable	Variable categories	Negative	Positive	(CI = 95%)	
Sex	Male	130	15	1	
	Female	249	27	1.06 (0.55–2.07)	
Age	18–24	111	14	1	
	25–34	156	16	1.23(0.58–2.62)	
	35–44	68	7	1.23(0.47–3.19)	
	≥45	44	5	1.11(0.38–3.27)	
Residence	Rural	83	2	5.61(1.33–23.69)^a^	4.89(1.15–20.82)^b^
	Urban	296	40	1	1
Marital status	Single	133	12	1	
	Married	239	28	0.77(0.38–1.56)	
	Divorced/Widowed	7	2	0.32(0.06–1.69)	
Educational status	Grade 1–8 & below	132	9	1	
	Grade 9–12	88	16	0.38(0.16–0.89)^c^	
	College and above	159	17	0.59(0.28–1.22)	
Time duration since gastritis started	< 1 month	62	5	1.59(0.59–4.29)	
	>1 to 3 months	106	10	1.36(0.63–2.91)	
	> 3 months	211	27	1	
Occupation	Employee	144	15	1	
	Non-employee**	235	27	0.91(0.47–1.76)	
Previous gastritis therapy	No	170	16	1.32(0.69–2.54)	
	Yes	209	26	1	
Presence of other disease(s)	No	284	29	1.34(0.67–2.68)	
	Yes	95	13	1	
Self-reported alcohol intake	Yes	214	23	1	
	No	165	19	0.93(0.49–1.77)	
Pain feeling period in the day	After meal	196	21	1	
	Persistent to the day	108	14	0.83(0.40–1.69)	
	Long interval b/n meals	75	7	1.15(0.47–2.81)	
Use of Flaxseed or Fenugreek	No	262	24	1.68 (0.88–3.21)	
	Yes	117	18	1	
Triple therapy regimen duration	10 day Amox+Clari +PPI	249	30	1	
	14 day Amox+Clari +PPI	130	12	1.31(0.65–2.63)	
Adverse drug effects	Yes	90	20	1	1
	No	289	22	2.92(1.52–5.59)^d^	2.80 (1.44–5.43)^e^

*SAT after HPET (Stool Antigen Test after H. pylori Eradication Therapy)

**without monthly salary

^a^p = 0.019

^b^p = 0.032

^c^p = 0.025

^d^p = 0.001

^e^p = 0.002

## Discussion

The aim of this study was to assess *H*. *pylori* eradication rate and determine the factors affecting eradication therapy. The overall *H*. *pylori* eradication rate of standard triple therapy was 90.02%. There are no similar studies conducted in the country to compare with the present result. However, varied rates have been reported from studies performed elsewhere. For example, rates similar with the present study (85–94%) are reported in some studies [23]. But, lower rates (61–77%) are also reported in other studies [24]. Adherence to therapy and resistance of *H*. *pylori* have been suggested to be the most important factors in *H*. *pylori* eradication therapy. Optimum eradication rate of *H*. *pylori* infection can only be achieved if adherence to drug therapy is higher in susceptible areas. It has been repeatedly reported that factors leading to poor adherence have paramount importance to determine successful treatment outcomes[25,26]. Thus, it is plausible to assume that the eradication rate differences could be associated with extent of adherence of patients to prescribed medications and/or local susceptibility pattern of *H*. *pylori* in the study areas. Considering these variability across regions, a meta-analysis has recommended that choice of antibiotics should be localized [27].

The standard triple therapy comprising of PPI, clarithromycin, and amoxicillin or metronidazole has become universal, as all of the consensus conferences and guidelines worldwide recommend this treatment for *H*. *pylori* eradication [28]. An eradication rate of over 90% has been regarded as the optimal eradication cut-off therapy for per-protocol analysis [29]. The effectiveness of *H*. *pylori* infection eradication therapy regimens, as per-protocol analysis, has been stratified based on the rate as excellent (>95%), good (91%-95%), borderline (85%-89%), and unacceptable (<85%) [30]. In view of this suggestion, the present eradication rate is closer to good, indicating that the therapy is effective. Better eradication rate in the present study could be explained by: i) higher dose of therapy in relation to lower mean body weight of the patients; ii) longer duration of therapy, 10–14 days compared to 7 days therapy; and iii) low rate (6%) of resistance to amoxicillin and complete susceptibility for clarithromycin [31]. According to a systematic review, worldwide prevalence of resistance to amoxicillin and clarithromycin was 11.2% and 17.2%, respectively [32], which is higher than the above mentioned figure reported in Ethiopians.

Of the possible variables assessed in the present study, only area of residence and self-reported adverse drug effects were factors that significantly affect *H*. *pylori* eradication rate. Patients living in rural areas were more likely to eradicate *H*. *pylori* infection than those living in urban areas. This is the first study to report the effect of residence on eradication and hence it is not possible to relate it with the literature. However, it could be associated with lower proportion of self-reported adverse drug effects in patients from rural area (17.5%) compared to urban area (28.3%). Obviously, eradication rate among patients with little or no adverse drug effects is higher than those who report more adverse drug effects, as there is better adherence with the former than the latter.

The percentage of self-reported adverse drug effects (26.1%) in the present study is higher than some (10–18%) [33–35] and lower than others (48–76%) [24,36] previously published studies. The difference could be due to several factors such as duration of triple therapy, socio-demographic differences of patients, pharmacogenetic variability among patients, drug combinations and possible interactions among these factors.

It is suggested that the efficacy of most *H*. *pylori* eradication regimens can be improved through use of potent acid secretion inhibitors, higher doses of antibiotics, and increasing duration of triple therapy [37, 38]. In this study, duration of triple therapy did not have influence on eradication rate. Likewise, no apparent difference in effectiveness was observed between omeprazole and pantoprazole-based therapy, confirming alternative use of the two drugs.

Age and sex were not significantly associated with eradication therapy as reported previously [39]. Mean age of patients (30.63 years) in this study is comparable with previously reported mean age (28.8 years)[40] in the same area, but lower than (40–53) studies done in other countries [36,41,42]. This indicates that the disease might be more prevalent in young adults. Percentage of female patients (65.6%) in the present study is comparable to previously reported prevalence of *H*. *pylori* infection among females in Ethiopia [43,44] as well as in other countries (62–65%) [34,41]. However, lower (28.6%) [36] and higher (75%) [42] prevalence in females has also been reported.

Alcohol did not affect eradication rate in this study and this is consistent with other studies [25,36]. There are different reports on effect of alcohol on *H*. *pylori* eradication, some reported as having effect on eradication, while others not. These differences could be due to differences in defining reported alcohol use and/or cultural differences of alcohol use in study populations.

Almost one-third (32.1%) of patients reported that they had taken diets traditionally believed to have healing effect on acid-pepsin disorder like Flaxseed or Fenugreek during therapy. In this study, use of either Flaxseed or Fenugreek had no significant effect on eradication rate. In line with this finding, diet was reported not to have effect on *H*. *pylori* eradication rate [45], although addition of fermented milk is reported to increase eradication [46].

Self-reported presence of other chronic diseases in this study was not a factor affecting eradication rate. Effect of other chronic diseases, such as diabetes mellitus, hypertension, chronic kidney disease, chronic liver disease, and chronic lung disease are thought to affect *H*. *pylori* eradication therapy occasionally, however, the results are inconsistent and the evidence is limited [47,48].

## Conclusion

*H*. *pylori* eradication rate found in the present study is within the recommended level for clinical practice, indicating that modifications made in the standard triple therapy observed in different healthcare institutions, at least, in the study area are not based on evidence. Emphasis to adverse drug effects of medications and area of residence of patients during counseling could have contribution in improving eradication rate. Extending duration of therapy from 10 to 14 days or traditional supplementation practice of Flaxseed or Fenugreek by patients at home might not ensure success in the eradication therapy. Further studies on antibiotic susceptibility of *H*. *pylori* should be done to evaluate resistance as a factor in affecting eradication rate.

## Supporting information

S1 File Consent form. (PDF)

S1 Table Sociodemographic data of *Helicobacter pylori* positive patients included in the study. (PDF)

S2 Table Response of *Helicobacter pylori* positive patients on first encounter. (PDF)

S3 Table Response of *Helicobacter pylori* positive patients on second encounter. (PDF)

S4 Table Format for laboratory result of patients on *H. pylori* eradication therapy research. (PDF)

S1 Dataset Raw underlying data. (SAV)
