# Supplementary material for: Helicobacter pylori eradication rate of standard triple therapy and factors affecting eradication rate at Bahir Dar city administration, Northwest Ethiopia: A prospective follow up study
Source: PLoS One. 2019 Jun 4;14(6):e0217645. doi: 10.1371/journal.pone.0217645 (PMC6548423; doi:10.1371/journal.pone.0217645)
Supplement: S1 File — (PDF) [file pone.0217645.s001.pdf]

S1 file

My name is Endalew Gebeyehu. I am the principal investigator of a research entitled "*H. pylori* eradication rate of standard triple therapy and factors affecting its eradication rate at Bahir Dar city administration, Northwest Ethiopia: A prospective follow up study". I am an academic staff at department of Pharmacology, College of Medicine and Health Sciences, Bahir Dar University and PhD candidate at department of Pharmacology and Clinical Practice, School of Pharmacy, Addis Ababa University.

This study has obtained ethical approval from Research Ethics Committee of College of Medicine and Health Sciences, Bahir Dar University. Your participation in this study is based on your voluntariness. If you agree to participate what is expected from you is your presence on appointment so that health professionals can collect relevant information related to *H. pylori* eradication therapy. Although patients undergoing *H. pylori* eradication therapy will be free of the infection after therapy the infection may exist due to different factors affecting eradication therapy. Assessing extent of eradication and the factors affecting eradication through findings obtained in this research could have paramount importance in improving *H. pylori* eradication therapy. We assure you that the confidentiality and privacy of the information collected will be kept through recording data anonymously and restricting data access.

I appreciate your participation in this study. Thank you!!

Name and signature of participant patients____________________________________.

Name and signature of principal investigator___________________________________.
