# Supplementary material for: Helicobacter pylori eradication rate of standard triple therapy and factors affecting eradication rate at Bahir Dar city administration, Northwest Ethiopia: A prospective follow up study
Source: PLoS One. 2019 Jun 4;14(6):e0217645. doi: 10.1371/journal.pone.0217645 (PMC6548423; doi:10.1371/journal.pone.0217645)
Supplement: S1 Table — (PDF) [file pone.0217645.s002.pdf]

S1 Table

S1 Table: Sociodemographic data

Sr. No	Questions	Response
100	Patient Card No: ________________	Code given_________________
101	Sex	☐ Male<br>☐ Female
102	Age	_______in years
103	Weight	________Kg
104	Height	________Meter
105	Address	Region___________________<br>Zone_____________________<br>Woreda___________________<br>Kebele______________________<br>Phone No:___________________
106	Residence	☐ Rural<br>☐ Urban
107	Marital status	☐ Single<br>☐ Married<br>☐ Divorced<br>☐ Widowed<br>☐ Separated
108	Religion	☐ Orthodox<br>☐ Protestant<br>☐ Muslim<br>☐ others, specify __________

109	What is your occupation?	☐ House wife<br>☐ Gov't Employee<br>☐ Private Employee<br>☐ Merchant<br>☐ Daily laborer<br>☐ 7. Others, specify________________
110	Your educational status is?	☐ Unable to read and write<br>☐ Read and write<br>☐ Primary education(1-8Th grade)<br>☐ Secondary education (9-12th grade)<br>☐ College and above
111	Ethnicity	☐ Amhara<br>☐ Tigrie<br>☐ Agew<br>☐ Oromo<br>☐ Guragie<br>☐ Others
112	Average monthly family income	in birr ________________
