# Supplementary material for: Helicobacter pylori eradication rate of standard triple therapy and factors affecting eradication rate at Bahir Dar city administration, Northwest Ethiopia: A prospective follow up study
Source: PLoS One. 2019 Jun 4;14(6):e0217645. doi: 10.1371/journal.pone.0217645 (PMC6548423; doi:10.1371/journal.pone.0217645)
Supplement: S2 Table — (PDF) [file pone.0217645.s003.pdf]

S2 table

S2 Table: Response of *H. pylori* positive patients on first encounter

Sr. No	Questions	Possible responses
201	Have you ever diagnosed with H pylori infection before this?	☐ Yes (old)<br>☐ No (New)
202	Have you taken triple therapy previously	☐ Yes<br>☐ No
203	When your current health problem started?	☐ Since this week<br>☐ Since last two weeks<br>☐ Since a month<br>☐ Since three months<br>☐ Since six months<br>☐ Since a year<br>☐ Since two years<br>☐ Before three years
204	Have you taken medications in the last two weeks?	☐ Yes (can you list___________)<br>☐ No
205	When you feel discomfort/pain	☐ After meal<br>☐ Before meal<br>☐ Persistently or Always<br>☐ At night
206	Which alcoholic drink(s) you had taken before you came for medical care? (more than one response)	☐ Traditional alcoholic drinks (Tella, Arekie, Teji)<br>☐ Bears<br>☐ Woin<br>☐ Wuski<br>☐ Others, specify _____________
207	Do you have history of other chronic illnesses? (more than one response is possible)	☐ Liver disease<br>☐ Kidney disease<br>☐ Diabetes<br>☐ Hypertension

		☐ Asthma<br>☐ Oher_____________
208	Do you smoke?	☐ Yes<br>☐ No
209	What traditional homemade remedies you took for the illness before you came here	______________________________<br>______________________________<br>______________________________
210	What modern medication other than the three drugs you took for the illness before you came here?	______________________________<br>______________________________<br>__________________________
