# Supplementary material for: Helicobacter pylori eradication rate of standard triple therapy and factors affecting eradication rate at Bahir Dar city administration, Northwest Ethiopia: A prospective follow up study
Source: PLoS One. 2019 Jun 4;14(6):e0217645. doi: 10.1371/journal.pone.0217645 (PMC6548423; doi:10.1371/journal.pone.0217645)
Supplement: S3 Table — (PDF) [file pone.0217645.s004.pdf]

S3 Table

S3 Table: Response of *H. pylori* positive patients on second first encounter

Sr. No	Questions	Possible responses
300	Can you give me your appointment card?	Card No: ________________
301	Could you stand on the balance here?	Patient weight:_____________
302	How much confident are you on your medication administration	☐ Surely complete (100)%<br>☐ Mostly (80%)<br>☐ Partially<br>☐ Somewhat impossible
303	What are the major adverse drug effect you encounter during therapy	________________________________<br>________________________________<br>________________________________<br>________________________________<br>________________________________
304	Have you used homemade remedies during therapy	☐ Yes (If yes what?___________)<br>☐ No
305	What you are currently feeling on your previous health problem	☐ I am feeling nothing<br>☐ Improved but still feeling<br>☐ Not improved at all
