# Supplementary material for: Helicobacter pylori eradication rate of standard triple therapy and factors affecting eradication rate at Bahir Dar city administration, Northwest Ethiopia: A prospective follow up study
Source: PLoS One. 2019 Jun 4;14(6):e0217645. doi: 10.1371/journal.pone.0217645 (PMC6548423; doi:10.1371/journal.pone.0217645)
Supplement: S4 Table — (PDF) [file pone.0217645.s005.pdf]

S4 Table

S4 Table: Format for laboratory result of patients on H. pylori eradication therapy research

SNo	Date of 1st test	Card No	ID Code	SAT before Rx	Regimen of triple therapy	Date of 2nd test	SAT test after Rx
